# α-Lipoic acid prevents the intestinal epithelial monolayer damage under heat stress conditions: model experiments in Caco-2 cells

**DOI:** 10.1007/s00394-017-1442-y

**Published:** 2017-03-27

**Authors:** Soheil Varasteh, Johanna Fink-Gremmels, Johan Garssen, Saskia Braber

**Affiliations:** 1 Division of Veterinary Pharmacology, Pharmacotherapy and Toxicology, Institute for Risk Assessment Sciences, Utrecht University, 3584 CM Utrecht, The Netherlands; 2 Division of Pharmacology, Faculty of Science, Utrecht Institute for Pharmaceutical Sciences, Utrecht University, Universiteitsweg 99, 3584 CG Utrecht, The Netherlands; 3 Nutricia Research, 3584 CT Utrecht, The Netherlands

**Keywords:** α-Lipoic acid, Heat stress, Intestinal integrity, Oxidative stress, Inflammatory response

## Abstract

**Purpose:**

Under conditions of high ambient temperatures and/or strenuous exercise, humans and animals experience considerable heat stress (HS) leading among others to intestinal epithelial damage through induction of cellular oxidative stress. The aim of this study was to characterize the effects of α-Lipoic Acid (ALA) on HS-induced intestinal epithelial injury using an in vitro Caco-2 cell model.

**Methods:**

A confluent monolayer of Caco-2 cells was pre-incubated with ALA (24 h) prior to control (37 °C) or HS conditions (42 °C) for 6 or 24 h and the expression of heat shock protein 70 (HSP70), heat shock factor-1 (HSF1), and the antioxidant Nrf2 were investigated. Intestinal integrity was determined by measuring transepithelial resistance, paracellular permeability, junctional complex reassembly, and E-cadherin expression and localization. Furthermore, cell proliferation was measured in an epithelial wound healing assay and the expression of the inflammatory markers cyclooxygenase-2 (COX-2) and transforming growth Factor-β (TGF-β) was evaluated.

**Results:**

ALA pretreatment increased the HSP70 mRNA and protein expression under HS conditions, but did not significantly modulate the HS-induced activation of HSF1. The HS-induced increase in Nrf2 gene expression as well as the Nrf2 nuclear translocation was impeded by ALA. Moreover, ALA prevented the HS-induced impairment of intestinal integrity. Cell proliferation under HS conditions was improved by ALA supplementation as demonstrated in an epithelial wound healing assay and ALA was able to affect the HS-induced inflammatory response by decreasing the COX-2 and TGF-β mRNA expression.

**Conclusions:**

ALA supplementation could prevent the disruption of intestinal epithelial integrity by enhancing epithelial cell proliferation, and reducing the inflammatory response under HS conditions in an in vitro Caco-2 cell model.

**Electronic supplementary material:**

The online version of this article (doi:10.1007/s00394-017-1442-y) contains supplementary material, which is available to authorized users.

## Introduction

α-Lipoic acid (ALA, 1,2-dithiolane-3-pentanoic acid) is present in all kinds of pro- and eukaryote cells and is considered to be one of the most potent cellular antioxidants. ALA exhibits free radical scavenging properties, regulates antioxidant enzymes, has metal-chelating capacity, interacts with other antioxidants (vitamin C and E) [1, 2], and maintains its antioxidant function in both oxidized (disulfide, oALA) and reduced (di-thiol; dihydrolipoic acid, DHLA) forms [2]. In turn, ALA has been suggested as a treatment for different pathologies associated with redox imbalances including diabetes, ischemia–reperfusion injury, and heavy metal poisoning [3–5]. Besides the antioxidant activities, more recent investigations have demonstrated that ALA has anti-inflammatory properties and the therapeutic potential of ALA has been described in various inflammatory disorders [6, 7]. Trivedi and Jena demonstrated the protective effect of ALA against gut hyperpermeability, which was associated with a reduction in the systemic inflammation through the modulation of various molecular targets such as NF-κB, COX-2, IL-17, STAT3, Nrf2, NADPH: quinone oxidoreductase-1, in a murine model of ulcerative colitis [8]. In vitro and in vivo investigations by Fan et al. also showed a protective effect of ALA on the intestinal barrier function, which could be related to its antioxidant effect and to the increase in the expression of tight junctions proteins [9]. It has been reported that oxidative stress activates mitogen-activated protein kinase (MAPK) and phosphatidyl inositol 3-kinase (PI3K) signaling pathways, which would lead to a decrease in the expression of tight junction proteins [10–13]. Therefore, it seems plausible that ALA prevents the disruption of intestinal integrity by maintaining the expression of tight junction proteins through a suppression of MAPK and PI3K activation [11, 14].

Heat stress (HS), experienced by humans and animals under conditions of high ambient temperatures and/or strenuous exercise, is known to disturb the balance between the production of reactive oxygen species (ROS) and the antioxidant defense system at the cellular level, resulting in oxidative stress [12]. A hallmark in the protection of cells against HS is the production of heat shock proteins (HSPs), acting as chaperones in the folding and refolding of cellular proteins [15]. HSPs genes are transcriptionally activated by heat shock factor-1 (HSF1) and the mechanism by which HSPs, in particular HSP70, maintain protein homeostasis during (heat) stress is the inhibition of the protein aggregation and misfolding, thus preventing their irreversible denaturation [15].

We and others have previously shown that dysfunction of the intestinal barrier can be caused by HS and can lead to increased intestinal permeability and corresponding inflammatory response [16–18]. Considering the beneficial effects of ALA as an antioxidant and its role in the protection of the intestinal barrier function, we hypothesized that ALA could prevent HS-induced cellular dysfunction and disruption of intestinal barrier integrity.

Hence, we determined the preventive effects of ALA against HS-induced intestinal epithelial damage, using a well-established epithelial colorectal adenocarcinoma (Caco-2) cell culture model and monitored the response to HS, measuring redox regulation, intestinal integrity, cell proliferation (wound healing assay), and the corresponding inflammatory response. The obtained in vitro results contribute to the understanding of the mechanisms of action of ALA and suggest that ALA is a promising candidate to be used as a food additive to increase the resilience to heat stress.

## Materials and methods

### Cell culture

Colorectal adenocarcinoma (Caco-2) cells, obtained from the American Type Tissue Collection (Code HTB-37) (Manassas, VA, USA, passages 5–19), were cultured as described previously [16]. Cells were seeded on 0.3 cm^2^ high pore density polyethylene terephthalate membrane transwell inserts with 0.4 µm pores (Falcon, BD Biosciences, USA) placed in a 24-well plate (0.3 × 10^5^ cells/transwell insert) and the transwell experiments were conducted after obtaining a differentiated confluent Caco-2 monolayer at day 17–19 of culturing [19]. For the wound healing and nuclear protein extraction assays, Caco-2 cells were seeded at a density of 1 × 10^5^ cells/well in 6-well plates and experiments were started after 10 and 17 days of culturing, respectively.

### ALA pretreatment and HS exposure

ALA was obtained from Sigma–Aldrich [(±)-α-Lipoic acid, St. Louis, MO, USA]. Prior to HS exposure, Caco-2 cells were pretreated with DMEM (control) or DMEM supplemented with different clinically relevant concentrations of ALA (15, 30, 60 µM) [20, 21] added to the apical and basolateral compartments for 24 h [22]. Thereafter, Caco-2 cells were subjected to 37 °C or HS conditions (42 °C) in a humidified atmosphere of 95% air and 5% CO_2_ for 6 or 24 h without changing the medium.

Overall, Caco-2 cells were stimulated with ALA for 30 h (24 + 6 h) or 48 h (24 + 24 h) depending on the assay.

For all mRNA expression studies, 6-h HS exposure was used based on our previous published results [16]. Due to the fast activation of HSF1 and Nrf2 under stress conditions, Western blot analysis of HSF1 and Nrf2 was also conducted after 6-h HS exposure. For all functional assays, TEER measurement, paracellular permeability assay, wound healing assay, as well as for protein expression of HSP70 and E-cadherin by Western blot analysis and immunofluorescence staining, Caco-2 cells were exposed to HS for 24 h [16, 23, 24].

Neither HS exposure for 24 h nor ALA pretreatment in the used concentration affected the Caco-2 cell viability measured by lactate dehydrogenase release (Supplementary Fig. 1).

### RNA extraction and quantitative Real-Time PCR (qRT-PCR)

The mRNA expression of different target genes [HSP70, nuclear factor erythroid 2-related factor 2 (Nrf2), cyclooxygenase-2 (COX-2), and transforming growth factor-β (TGF-β)] was measured by qRT-PCR in Caco-2 cells pretreated with different concentrations of ALA for 24 h followed by exposure to HS for 6 h. RNA extraction, cDNA synthesis, and qRT-PCR analysis were performed according to a previously described protocol [16]. Forward and reverse primers (Eurogentec, Seraing, Belgium) with corresponding annealing temperatures are represented in Supplementary Fig. 2. The mRNA quantity was calculated relative to the expression of β-actin reference gene.

### Western blot (WB) analysis

Caco-2 cells pretreated with different ALA concentrations for 24 h and exposed to HS for 6 h (HSF1, Nrf2) or 24 h (HSP70, E-cadherin) were lysed using 50 µl RIPA lysis buffer (Thermo Scientific, Rockford, IL, USA) containing protease inhibitors (Roche Applied Science, Penzberg, Germany). Lysates were normalized for protein content and WB analysis was conducted as described previously [17] using primary antibodies against HSF1 (1:1000; Cell Signaling, Danvers, MA, USA), HSP70 (1:1000; Enzo Life Sciences, Farmingdale, NY, USA) or E-cadherin (1:1000, eBioscience, San Diego, CA, USA), and β-actin antibody (1:4000; Cell Signaling) for equality of sample loading. For the detection of Nrf2, the nuclear protein extracts were separated using NE-PER Nuclear and Cytoplasmic Extraction Reagents based on the manufacturer’s instructions (Pierce, Rockford, IL, USA). Nuclear protein concentrations were measured, normalized, and the WB analysis was conducted using primary antibodies against Nrf2 (1:1000; Cell Signaling) and Lamin A (1:1000; Cell Signaling) for equality of loading. Digital images were obtained with ChemiDoc™ MP imager (Bio-Rad Laboratories Inc.) and signal intensities were quantified using the ImageJ 1.47 software.

The protein expression was normalized with β-actin or Lamin A (for nuclear proteins) and expressed as mean fold change in relation to the control group.

### Immunofluorescence staining

Caco-2 cells were pretreated with 60 µM ALA for 24 h and exposed to HS for 24 h. Cellular localization of HSF1 and E-cadherin was assessed by an immunofluorescence staining as described previously [16]. Briefly, after HS exposure, cells were fixed with 10% formalin, washed with PBS, and permeabilized with PBS containing 0.1% Triton-X-100. After blocking in 5% serum, cells were incubated with anti-HSF1 (1:100, Cell Signaling, Danvers, MA, USA) and anti-E-cadherin (1:50, eBioscience) antibodies for 2 h at room temperature followed by incubation with Alexa Fluor conjugated secondary antibodies (Invitrogen). After a nuclear counterstaining with Hoechst 33,342 (1:2000; Invitrogen), the inserts were mounted with FluorSave^™^ Reagent (Calbiochem) and immune-localization of HSF1 and E-cadherin was determined with a Nikon Eclipse TE2000-U microscope equipped with a Nikon Digital Sight DS-U1 camera using an 40× objective. HSF1-immunostained slides were also examined by a Leica TCS SPE-II confocal laser scanning microscope on a DMI4000 (Leica Microsystems, Wetzlar, Germany); images were acquired with an oilimmersion objective (63×) and assembled using ImageJ 1.47 software.

### Transepithelial electrical resistance (TEER) measurement

The integrity of the transwell-grown Caco-2 monolayers pretreated with ALA (24 h) and exposed to HS (24 h) was determined by measuring TEER using a Millicell-ERS voltohmeter (Millipore, Temecular, CA, USA). Average TEER values prior to the start of the experiment were in the range of 400 ± 30 Ω cm^2^. Results are expressed as a percentage of initial value.

### Paracellular permeability assay

The paracellular permeability across the Caco-2 monolayer was investigated by measuring the lucifer yellow (LY, 0.457 kDa, 20 µg/ml; Sigma) flux. LY was added to the apical compartment (4 h prior to the end of HS exposure) for 4 h and the fluorescence intensity of LY in the basolateral compartment was measured by fluorometer (FLUOstar OPTIMA, Offenburg, Germany) at excitation and emission wavelengths of 410 and 520 nm, respectively.

### Calcium switch assay

Caco-2 cells grown on inserts were pretreated with different ALA concentrations added to the apical and basolateral compartment for 24 h. Subsequently, cells were washed with PBS and cell–cell contacts were disrupted by incubation with 2 mM EGTA (Sigma) in calcium- and magnesium-free HBSS (Gibco, Invitrogen, CA, USA) for 20 min. After the incubation, HBSS-EGTA was removed, cells were rinsed, and cell–cell junctions were allowed to re-establish by incubation with either complete cell culture DMEM (containing 2 mM CaCl_2_) or in DMEM supplemented with ALA concentrations. TEER values were measured at different time points (0, 2, 4, 6, 8 h) during this recovery period [19].

### Wound healing assay

Post-confluent Caco-2 monolayers, seeded in 6-well plates (1 × 10^5^ cells/well), were pretreated with different concentrations of ALA for 24 h. The wound healing assay was conducted as previously described [25]. Briefly, standardized wounds were created in Caco-2 monolayers by scratching with a 200-µl pipette tip (Greiner Bio, Germany) across the maximum diameter of each well. Thereafter, detached cells were removed by washing with PBS and the cells were incubated with DMEM or DMEM supplemented with ALA under control or HS conditions for 24 h. Immediately after scratching as well as 24 h after scratching, phase contrast images were acquired with the Olympus CKX41 microscope (Olympus Co. Ltd., Tokyo, Japan) and wound widths were measured using digital imaging system software (Leica Application Suite V4.2). Data were calculated as percentages of wound area relative to the initial wound width.

### Statistical analysis

Results are expressed as means ± SEMs of 3 independent experiments (*n* = 3), each performed in triplicate. All data were tested for normality of distribution using the D’Agostino & Pearson omnibus normality test. Analyses were performed by using GraphPad Prism (version 6.05) (GraphPad, La Jolla, CA, USA). Differences between groups were statistically determined by using Two-way analysis of variance (ANOVA), with Bonferroni post hoc test. For the calcium switch assay (single factor experiment), one-way ANOVA was performed. Results are considered statistically significant when *P* < 0.05.

## Results

### HS upregulates the HSF1 protein expression and induces HSF1 nuclear granules

**Fig. 1:**
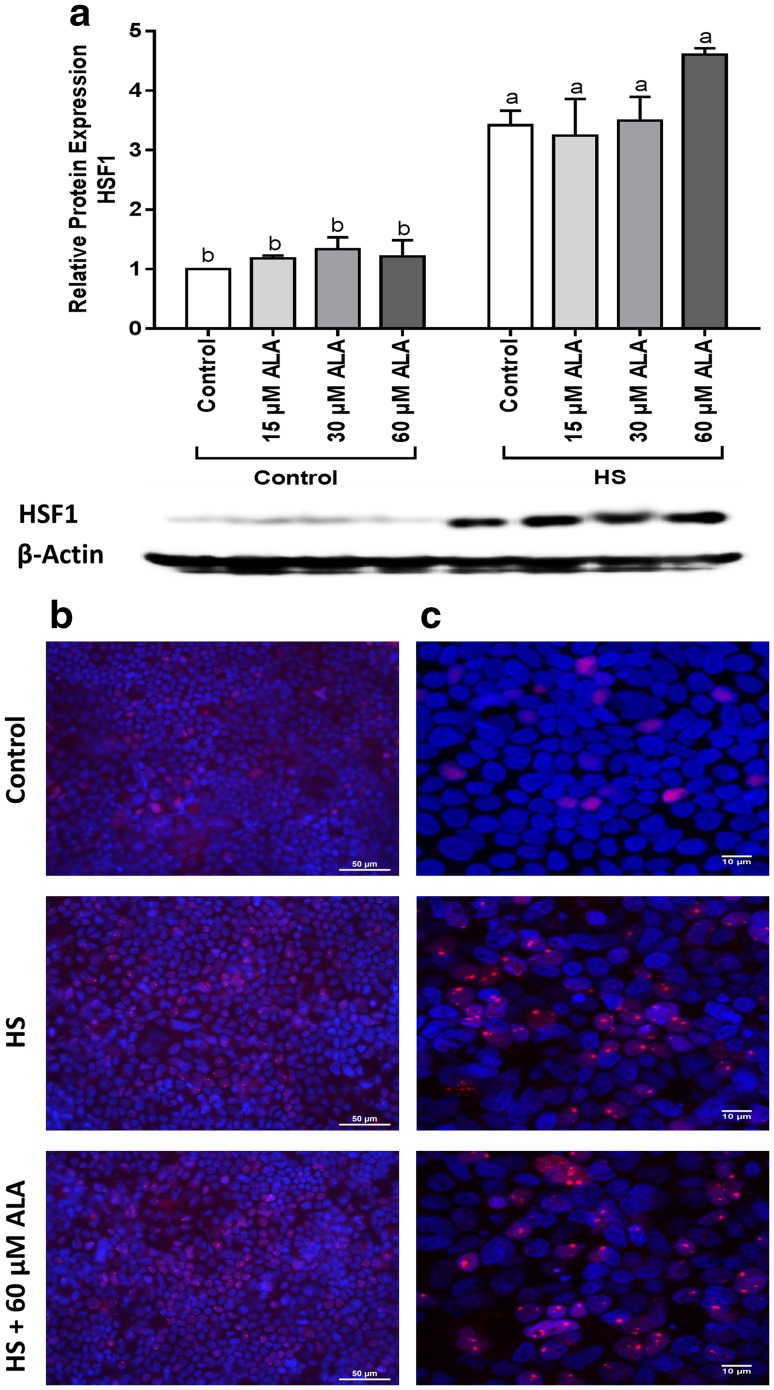
HS upregulates the HSF1 protein expression and induces HSF1 nuclear granules. Caco-2 cells grown on inserts and pretreated with ALA (24 h) were exposed to HS (42 °C, 6 h). HSF1 protein expression (normalized with β-actin) relative to unstimulated cells evaluated by WB analysis (**a**), is expressed as mean ± SEM of three independent experiments. *Different lower case letters* denote significant differences among groups. Localization of HSF1 was visualized by immunofluorescence staining. Objective ×40 (**b**) and ×63 (**c**)

The HSF1 protein expression significantly increased after 6-h HS exposure. Although pretreatment with ALA did not significantly change the expression of HSF1, HSF1 protein levels tended to increase in heat-exposed Caco-2 cells incubated with ALA (Fig. 1a). The immunofluorescence staining revealed that exposure to HS resulted in HSF1-granule formation, whereas no clear effect of ALA treatment was observed (Fig. 1b, c).

### ALA enhances the expression of HS-induced HSP70 in mRNA and protein level

**Fig. 2:**
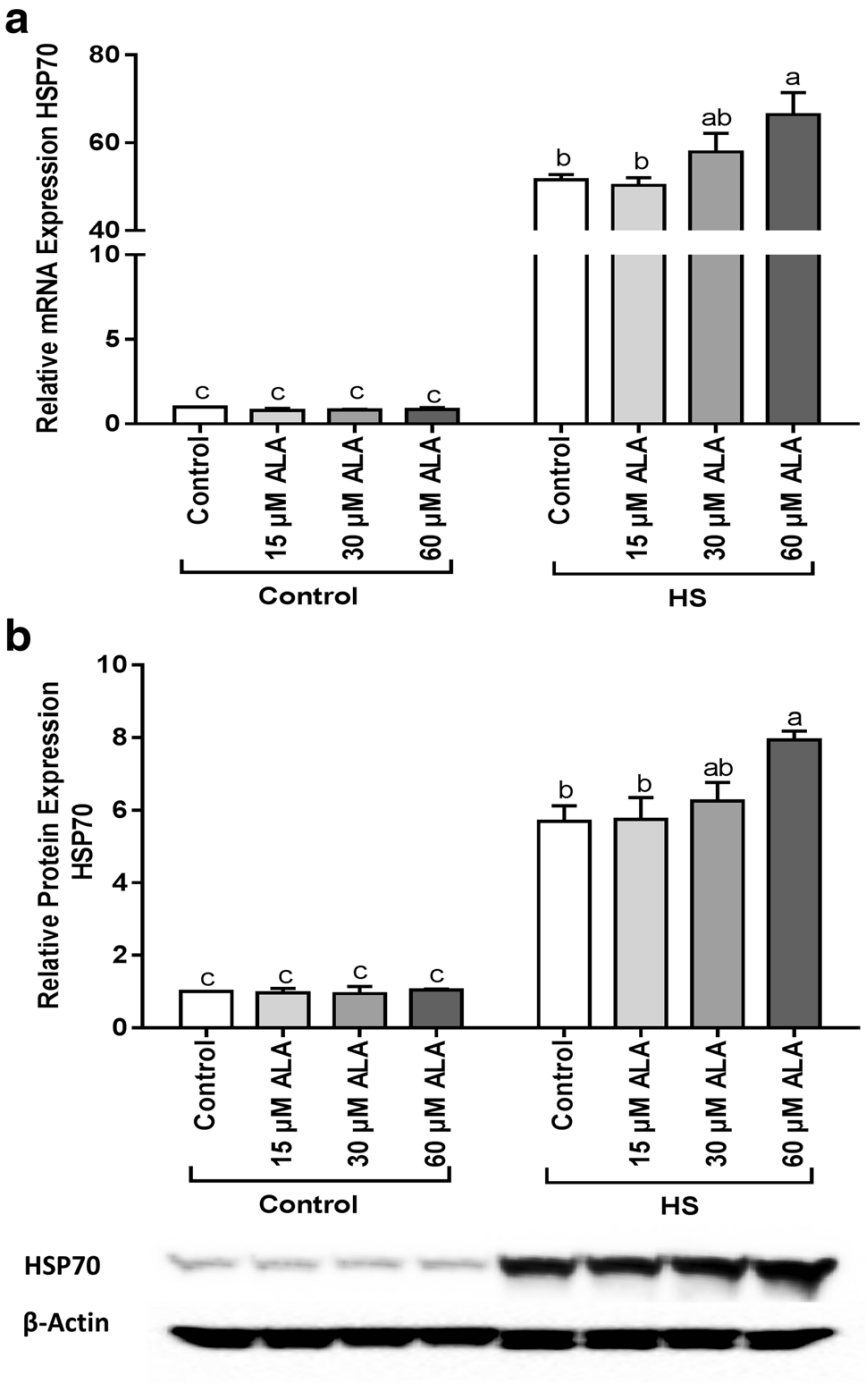
ALA increases the HSP70 expression under HS conditions. Caco-2 cells grown on inserts and pretreated with ALA (24h) were exposed to HS (42 °C) for 6 h (qRT-PCR) or 24 h (WB) to evaluate the expression of HSP70 in mRNA (**a**) and protein levels (**b**). Results are expressed as mRNA expression or protein expression (normalized with β-actin) relative to unstimulated cells as mean ± SEM of three independent experiments. *Different lower case letters* denote significant differences among groups

Pretreatment of Caco-2 cells with ALA enhanced the HS-induced upregulation of HSP70 in mRNA (Fig. 2a) and protein level (Fig. 2b). Significant changes were only observed at the highest ALA concentration (60 µM), whereas lower concentrations of ALA (15 and 30 µM) did not significantly increase the expression of HSP70 in mRNA or protein levels.

### ALA modulated Nrf2 expression and translocation in Caco-2 cells exposed to HS

**Fig. 3:**
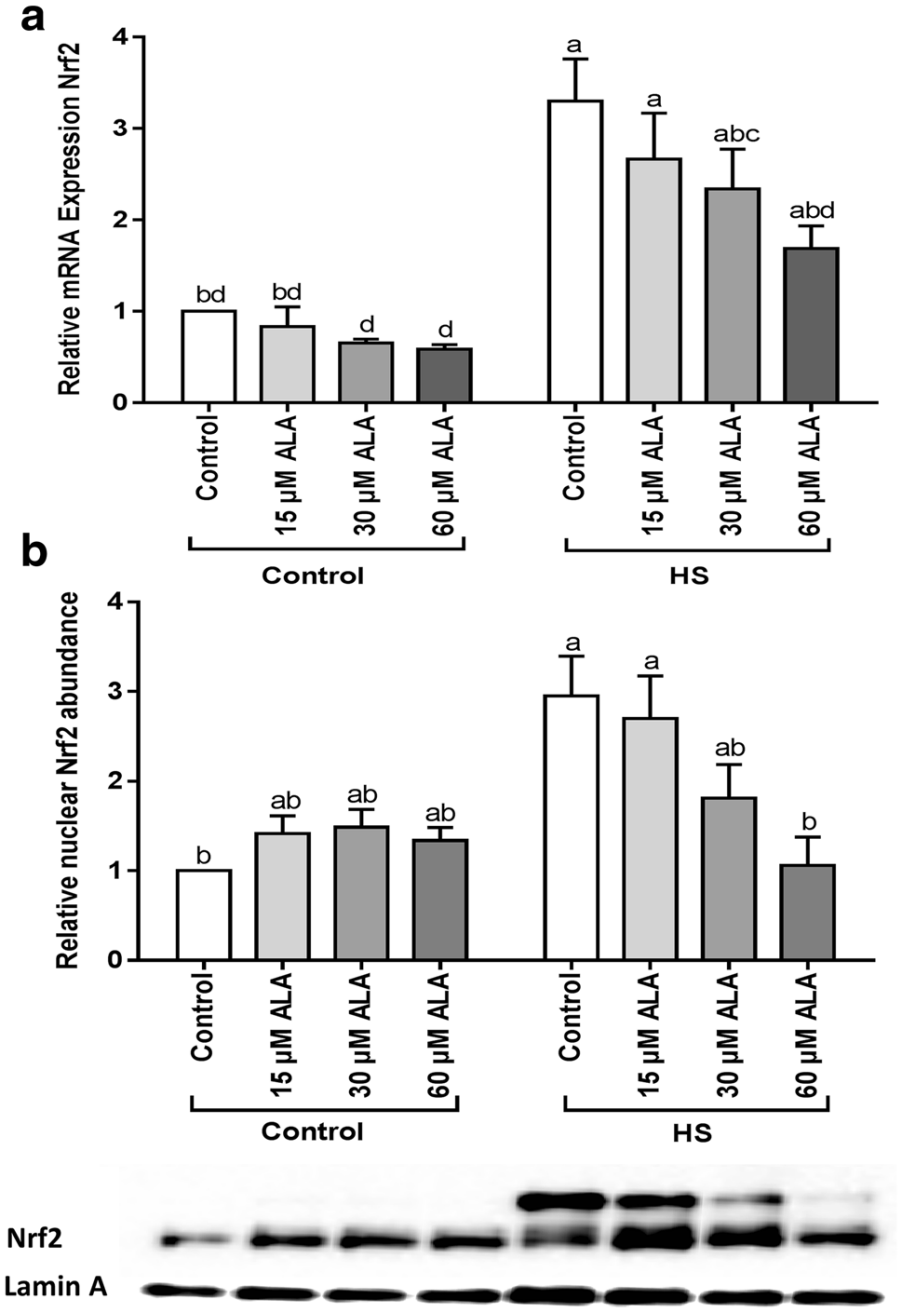
ALA prevents the HS-induced expression and nuclear translocation of Nrf2. Caco-2 cells grown on inserts (qRT-PCR) or 6-well plates (WB) and pretreated with ALA (24 h) were exposed to HS (42 °C) for 6 h. Results are expressed as mRNA expression (normalized with β-actin) (**a**) and nuclear abundance (normalized with Lamin A) (**b**) relative to unstimulated cells as mean ± SEM of three independent experiments. *Different lower case letters* denote significant differences among groups

At the transcriptional level, Nrf2 was significantly upregulated under HS conditions and this effect was mitigated by ALA (Fig. 3a). Upon exposure to HS, the abundance of Nrf2 protein in the nucleus was markedly increased, and this effect was mitigated by 60 µM ALA (Fig. 3b). Exposure to ALA under control conditions slightly increased the Nrf2 protein levels in the nuclei (Fig. 3b). Moreover, ROS measurements showed that HS-induced ROS generation. ALA treatment slightly, but not significantly, increased ROS levels under control as well as HS conditions (Supplementary Fig. 3).

### ALA prevents the HS-induced disruption of the intestinal epithelial integrity

**Fig. 4:**
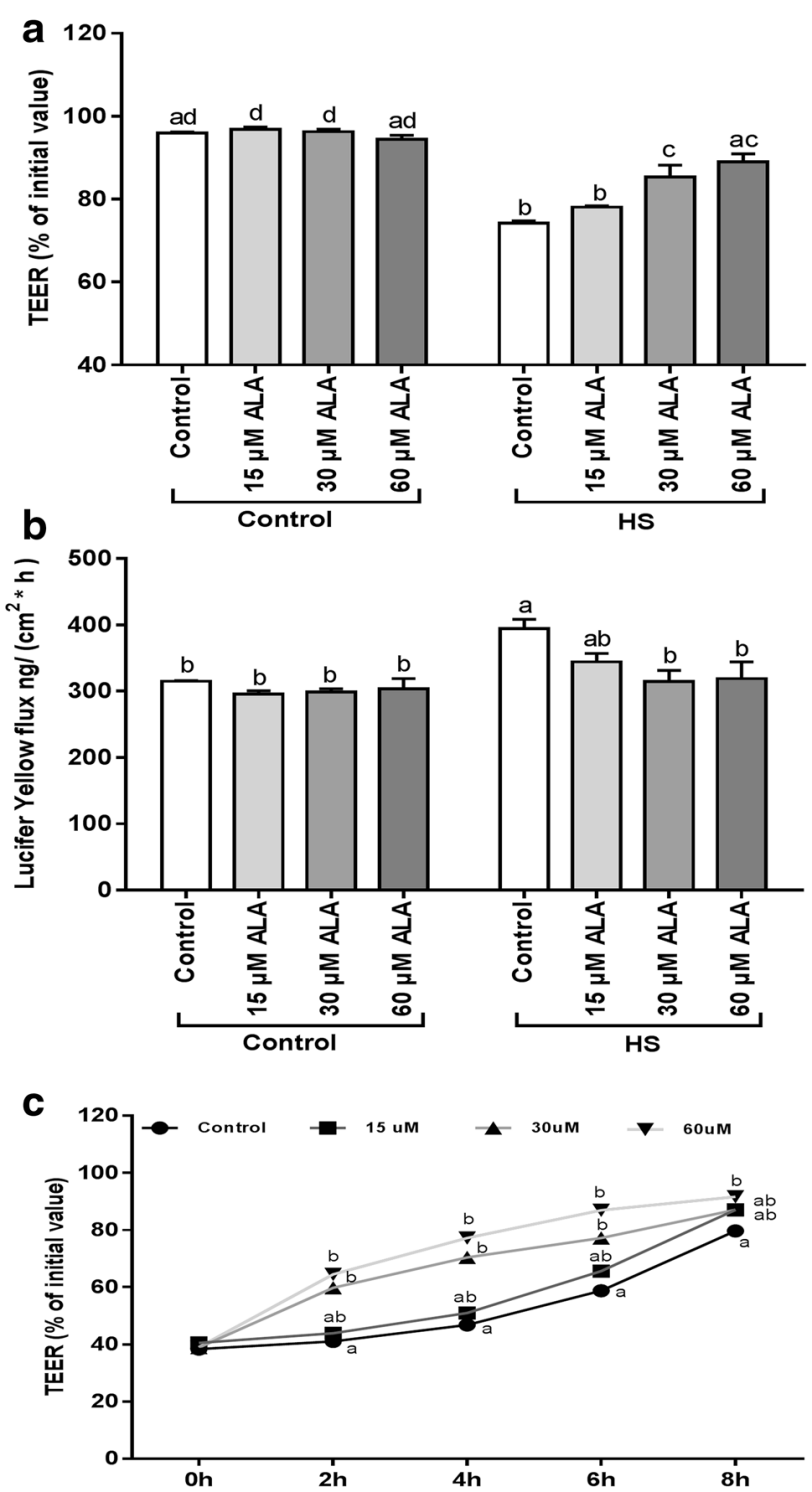
ALA prevents the HS-induced disruption of epithelial integrity and accelerates the JC reassembly. Caco-2 cells grown on inserts were pretreated with ALA (24 h) prior to HS exposure (42 °C) and TEER (**a**) and LY transport (**b**) across the Caco-2 monolayer was measured after 24 h exposure to HS. For the calcium switch assay, Caco-2 cells were pretreated with ALA (24 h) before calcium deprivation and TEER was measured during recovery (0, 2, 4, 6, 8 h) in medium supplemented with ALA (**c**). Data are expressed as a percentage of initial value (TEER, calcium switch assay) or in the amount LY transported [ng/(cm^2^ × h)] detected in the basolateral compartment of transwell inserts as mean ± SEM of three independent experiments. *Different lower case letters* denote significant differences among groups

The effect of ALA pretreatment on the HS-induced disruption of the epithelial barrier integrity was monitored by measuring the TEER values and LY flux. As shown in Fig. 4a, the decrease in TEER values induced by HS was significantly modulated by pretreatment with 30 and 60 µM ALA. In agreement with the TEER values, the HS-induced increase in LY flux across the Caco-2 monolayer was significantly prevented by 30 and 60 µM ALA pretreatment (Fig. 4b). Incubation of Caco-2 cells with 15 µM ALA did not significantly alter the HS-induced TEER decrease and LY permeability (Fig. 4).

### ALA facilitates the reassembly of junctional complexes (JC) after calcium deprivation

To examine the effect of ALA on the JC of the Caco-2 monolayer under dynamic disassembly/reassembly conditions, a calcium switch assay was performed. ALA concentrations of 30 and 60 µM significantly enhanced the reassembly of JC during 6 and 8 h, respectively, observed by TEER measurements over a period of 8 h after a temporary calcium deprivation (Fig. 4c), whereas 15 µM ALA did not have a remarkable effect (Fig. 4c).

### ALA partly prevents the HS-induced disturbance of E-cadherin expression

**Fig. 5:**
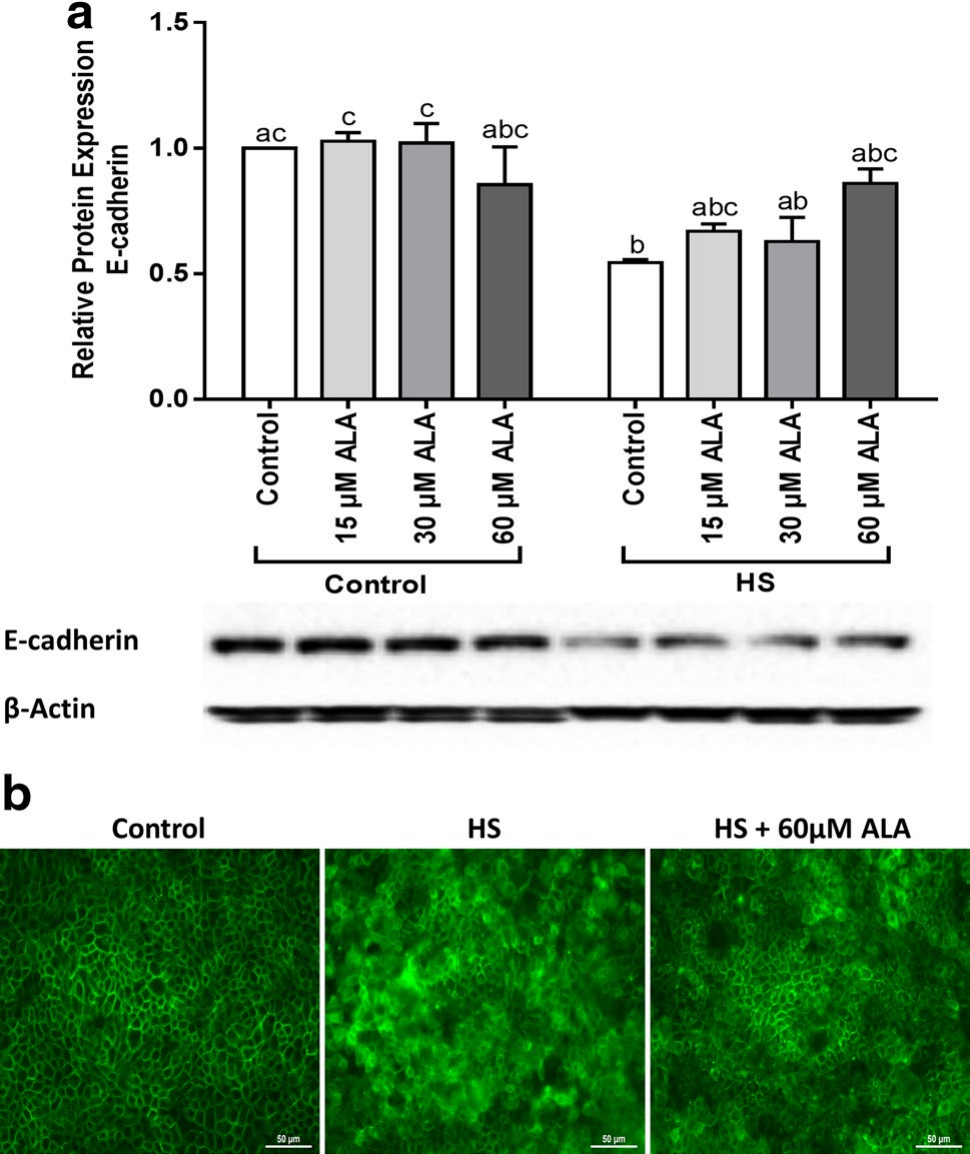
ALA partly prevents the HS-induced disturbance of E-cadherin expression. Caco-2 cells grown on inserts and pretreated with ALA (24 h) were exposed to HS (42 °C) for 24 h to evaluate the E-cadherin protein expression (**a**) and cellular distribution (**b**). For the WB analysis, results are expressed as protein expression (normalized with β-actin) relative to unstimulated cells as mean ± SEM of three independent experiments. *Different lower case letters* denote significant differences among groups. Localization of E-cadherin was evaluated by immunofluorescence staining (Objective, ×40)

Western blot analysis showed that exposure of Caco-2 cells to HS for 24 h resulted in significant decrease in E-cadherin protein level, which was partly, but not significantly, prevented by pretreatment with different ALA concentrations (Fig. 5a). The immunofluorescence staining of the E-cadherin distribution is depicted in Fig. 5b. In control cells, E-cadherin is localized at the cell membrane and appeared as belt-like structure around each cell. HS disrupted the distribution pattern of E-cadherin, whereas pretreatment with 60 µM ALA hampered this HS-induced irregular distribution of E-cadherin.

### ALA stimulates the restitution of intestinal epithelial wound healing

**Fig. 6:**
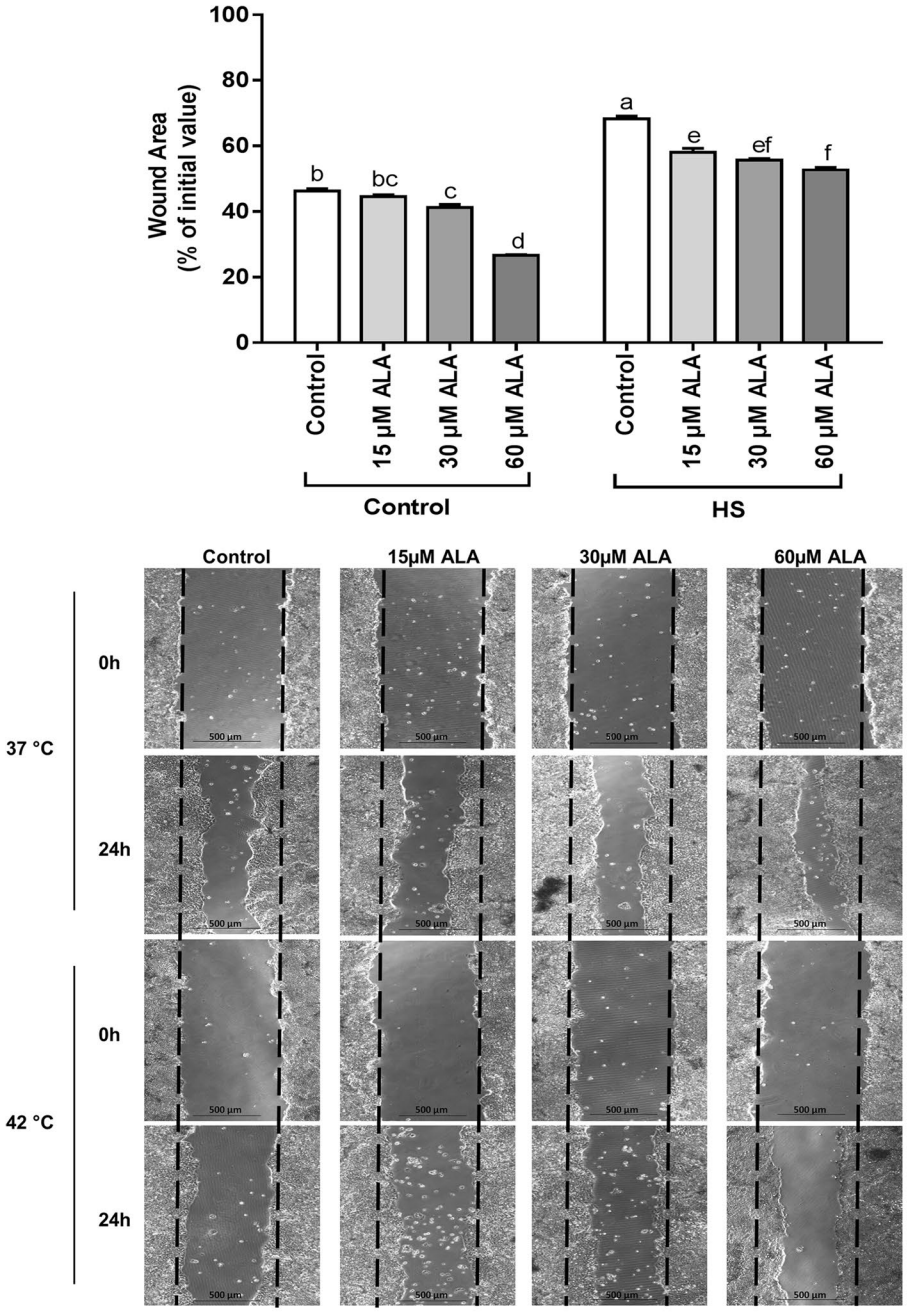
ALA stimulates the restitution of epithelial wound healing. Confluent Caco-2 cells grown in 6-well plates and pretreated with ALA (24 h) were scratched with a 200-µl pipette tip and were exposed to control or HS (42 °C) conditions for 24 h. Phase contrast images were acquired immediately after scratching (0 h) and 24 h thereafter. Wound widths are expressed as percentage of initial value as mean ± SEM of three independent experiments. *Different lower case letters* denote significant differences among groups

Pretreatment of the Caco-2 cell monolayers with 30 and 60 µM ALA significantly enhanced the wound healing at 37 °C. Exposure to HS caused a slower wound area closure compared to control cells at 37 °C, whereas the different concentrations of ALA (15, 30, and 60 µM) significantly stimulated wound healing under HS conditions (Fig. 6).

### ALA prevents the HS-induced upregulation of COX-2 and TGF-β mRNA expression

**Fig. 7:**
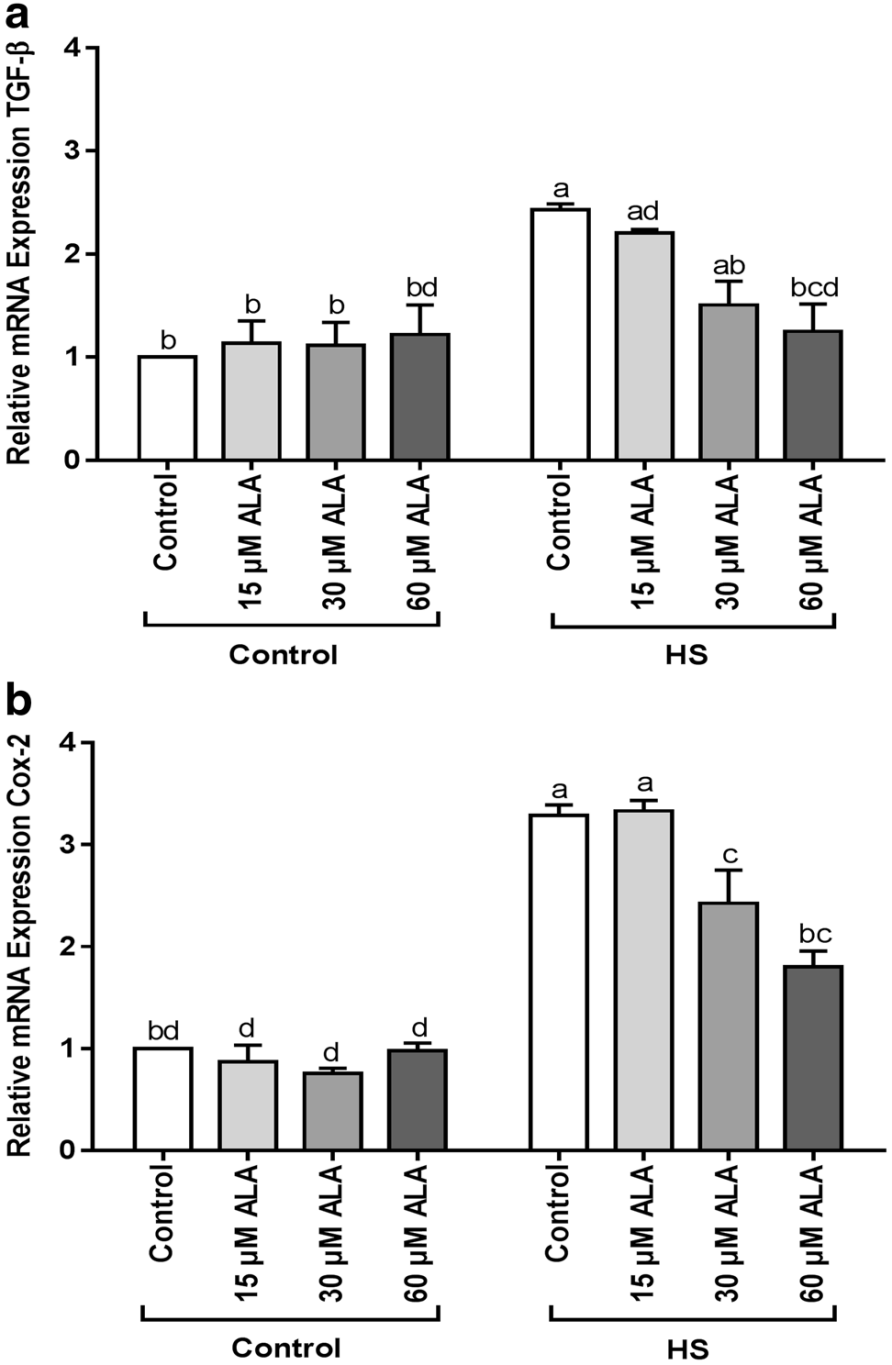
ALA prevents the HS-induced upregulation of TGF-β and COX-2 mRNA expression. Caco-2 cells grown on inserts and pretreated with ALA (24 h) were exposed to HS (42 °C, 6h) to evaluate the mRNA expression of TGF-β and COX-2 (qRT-PCR). Results are expressed as mRNA expression (normalized with β-actin) relative to unstimulated cells as mean ± SEM of three independent experiments. *Different lower case letters* denote significant differences among groups

Heat exposure significantly induced the TGF-β and COX-2 mRNA expression. Pretreatment with 60 µM ALA prevented the upregulation of TGF-β (Fig. 7a), whereas 30 and 60 µM ALA were effective in preventing the HS-induced COX-2 upregulation (Fig. 7b).

## Discussion

α-Lipoic acid is considered as one of the most potent cellular antioxidants and the chemical reactivity of ALA is mainly dependent on its dithiolane ring. The oxidized and reduced ALA forms (oALA and DHLA) create a potent redox couple, which is also called the “universal antioxidant” [1]. ALA participates in regenerating other antioxidants and, unlike ascorbic acid, DHLA is not destroyed by scavenging free radicals, but can be recycled from ALA. When ALA is administered in the diet, it accumulates in several tissues and a substantial part converts to DHLA via lipoamide dehydrogenase after ingestion. The cellular reduction of ALA to DHLA is accomplished by NAD(P)H-driven enzymes such as thioredoxin reductases [5].

ALA is recommended as a potential intervention strategy for pathologies associated with oxidative stress, and also exerts anti-inflammatory properties and is known to protect the intestinal barrier [7–9, 26]. We and others showed that HS can induce oxidative stress and lead to intestinal barrier disruption and inflammation [16–18]. Hence, the aim of this study was to investigate the potential beneficial effects of ALA against HS-induced intestinal epithelial injury using an in vitro Caco-2 cell model.

A heat shock response is initiated by activation of HSF1 transcription factor and upregulation of HSPs to promote cell adaptation and survival under a wide range of proteotoxic stressors, including thermal stress [27]. Therefore, in the current study, the effect of HS on the expression of HSF1 and HSP70 was investigated. Our results show that under HS conditions, HSF1 and HSP70 expression levels were significantly higher in comparison with the control group. Immunolocalization of HSF1 showed granule formation in the nucleus of the cells after HS exposure. It is described that HSF1 granules are transiently formed when heat shock genes are transcriptionally expressed, and quickly disappear after attenuation of HS-induced gene transcription [27]. Under HS conditions, pretreatment with ALA slightly but not significantly increased the HSF1 protein expression, whereas elevated mRNA and protein expression of HSP70 were observed. Although HSF1 mediates the upregulation of HSP70, overexpression of HSP70 can negatively regulate HSF1 transcriptional activity as described by Shi et al. [28]. There are already some indications that ALA might modulate the heat shock response by inducing HSPs or HSF1 [29–31]. For example, Oksala et al. described an increase in HSF1 mRNA expression in the kidney of both diabetic and nondiabetic rats after ALA supplementation [29]. Furthermore, enhanced recovery levels of inducible HSP70 in the muscle of horses were observed after ALA supplementation, which may increase oxidative capacity and support tissue protection and adaptation [32]. ALA can also induce the expression of other HSPs, including HSP25 (muscle), HSP60 (liver), HSP72 (kidney, muscle), and HSP90 (kidney) [29, 31–33].

The capability of ALA to modulate HSP expression has been shown to prevent oxidative injury [4]. To determine the effect of ALA on the HS-induced oxidative stress response, Nrf2 mRNA expression as well as nuclear translocation and ROS production was measured. ALA may act as a pro-oxidant, which triggers the Nrf2-dependent transcriptional activity by forming lipoyl-cysteinyl mixed disulfides on Keap1, a protein which suppresses Nrf2 in the cytoplasm [5, 34]. Our results clearly demonstrate that HS increases the mRNA expression and nuclear translocation of Nrf2. In turn, after pretreatment with ALA, the mRNA expression and nuclear translocation of Nrf2 is decreased under HS conditions. To defeat the HS-induced oxidative stress, Nrf2 dissociates from Keap1, translocates to the nucleus, and transactivates the expression of several cytoprotective genes such as glutathione and heme oxygenase-1 (HO-1) to enhance cell survival [35, 36]. In our experiments, ALA treatment only slightly but not significantly increased the HO-1 mRNA expression under HS conditions (Supplementary Fig. 4).

Supporting the previously described role of ROS generation in a HS-induced oxidative stress response [37], we could show a significant increase in ROS generation induced by HS. A slight, but not significant induction of ROS was demonstrated after ALA pretreatment under control as well as HS conditions. Although excessive ROS production is related to oxidative damage and cell death, available evidence suggests that a moderate increase in ROS levels, induced by pro-oxidants including ALA, is positively correlated with enhanced cell survival and resilience to oxidative damage [34, 38]. To unravel the underlying mechanism, Jiang et al. showed that ALA pretreatment in H9c2 cells attenuates cell damage through activation of Akt/Gsk-3β signaling in a ROS-related manner. Blockade of AKT activation as well as the inhibition of ROS production abrogates the cytoprotection induced by ALA [39].

Since ROS levels were not significantly affected by ALA under HS conditions, there might be also ROS-independent pathways involved in the regulation of Nrf2. It has been suggested that different protein kinases including casein kinase-2 and phosphoinositide-3-kinase could be involved in this ROS-independent activation of Nrf2 [40, 41]. Another possible mechanism could be related to the maintenance of cellular homeostasis induced by HSP70 upregulation. Guo et al. showed that HSP70 significantly increased the glutathione-related enzymes to preserve the cellular redox balance [42]. However, the exact mechanisms involved in the ROS-independent regulation of Nrf2 remains to be further elucidated.

Our previous in vitro and in vivo investigations showed that induction of HS is associated with disruption of intestinal integrity, which was linked mainly to alterations in E-cadherin protein expression and localization [16, 17]. This study indicates that ALA prevents the HS-induced decline in TEER levels in a concentration-dependent manner and this effect is confirmed by a reduced LY flux across the intestinal epithelial monolayer. The preventive effect of ALA against the HS-induced delocalization of E-cadherin might be related to the redox balance stabilized by ALA. It has been suggested that oxidative stress can induce a tyrosine kinase-dependent dissociation of E-cadherin-β-catenin and occludin-ZO1 complexes, which leads to their redistribution and disruption of barrier integrity [43]. Another possible mechanism is the known upregulation of HSP70 which is pivotal in preserving the barrier function under stress conditions [44]. Recently, an essential role of HSP-mediated cytoskeletal repair in a Caco-2 model of celiac disease was observed, as a shift of HSP70 from the cytoplasmic fraction into the cytoskeletal fraction of Caco-2 cells, resulted in the maintenance of barrier integrity through stabilization of E-cadherin protein [45]. Additionally, we observed that ALA supports the acceleration of the junctional complex after calcium deprivation under thermal neutral conditions. Although the underlying mechanism is not fully understood, it has been previously reported that ALA stimulates the recovery of the intestinal epithelial architecture by increasing the mRNA and protein expression of the tight junction proteins occludin and ZO-1 in a model system of post-weaning diarrhea in rats. These findings were confirmed by in vitro studies with IEC-6 intestinal epithelial cells [9]. However, the mRNA and protein expression of different tight junction proteins (claudin-1,-3 and -4, occludin, and ZO-1) were not affected in the current Caco-2 cell model as previously described by our group [16].

HS-induced morphological damage to the intestinal mucosa is associated with villi denaturation [46]. To mimic this type of injury, we conducted a wound healing assay. As ALA accelerated the wound healing, these findings suggest that re-epithelialization of the intestinal epithelial monolayer under thermal neutral and HS conditions can be expected. In in vivo experiments in rats, Ma et al. demonstrated that supplementation of the diet with ALA improves the morphology of the small intestine damaged by glycinin [6]. Redox-regulated processes are relevant to cell proliferation and wound healing [47] and depending on the concentration, ROS is described to stimulate epithelial cell proliferation and migration by promoting the phosphorylation of ERK1/2 and the expression of cyclin D1 [48]. Furthermore, the role of induced HSP70 expression should also be taken into account in the improved wound healing process by ALA, since HSPs suppress misfolding of proteins, thereby preventing cell proliferation arrest [49].

It has previously been reported that TGF-β signaling is involved in the inhibition of epithelial cell proliferation, failure in wound healing and the process of fibrosis [50]. Therefore, the mRNA expression of TGF-β was investigated in the HS-challenged Caco-2 cells. Our results show that ALA pretreatment was able to abolish the upregulation of TGF-β under HS conditions in a concentration-dependent manner and it can be suggested that ALA inhibits p38 MAPK, which is involved in regulation of TGF-β mRNA expression [51].

We also investigated the modulatory effect of ALA on the mRNA expression of COX-2, as an indicator of the inflammatory responses. Rossi et al. showed that HSF1, induced by HS exposure, binds to the COX-2 promoter and triggers the upregulation of COX-2 [52]. It has also been verified that oxidative stress is an important factor for the induction of COX-2 [53]. In the current study, ALA prevented the HS-induced upregulation of COX-2 mRNA expression. Considering the fact that COX-2 is a well-known NF-kB target gene [54], it is likely that ALA treatment prevented the COX-2 upregulation through NF-kB repression [1, 55]. In addition, HS exposure did not significantly increase the IL-8 release by these Caco-2 cells (data not shown).

The concentrations of ALA used in this study were lower than the ALA concentrations used in several other in vitro studies (tested concentrations in a range of 300–500 µm) [39, 56]. However, the ALA concentration, which will reach the intestinal epithelial cells under in vivo conditions cannot be exactly predicted and hence the optimal ALA dose to combat HS-induced disturbance of the intestinal epithelial monolayer, remains to be determined, but the selected ALA concentrations seem not to induce any clinical adverse effects [2, 57].

## Conclusions (summarized in Fig. 8)

**Fig. 8:**
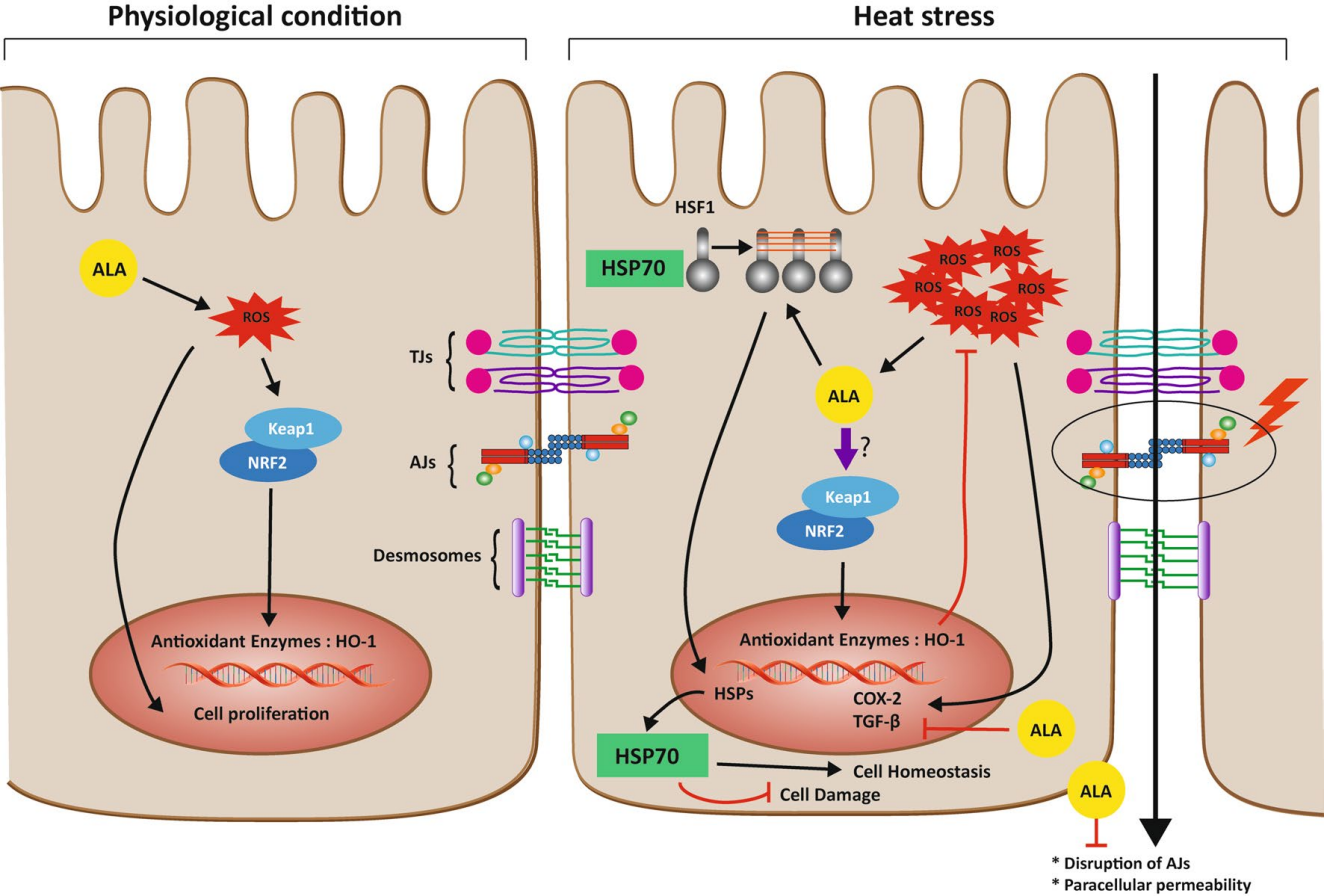
Proposed mechanism by which ALA regulates intestinal epithelial cells under HS conditions. Under physiological conditions, ALA induces a slight ROS production, which could trigger dissociation of Nrf2 from Keap1 and Nrf2 translocation to the nucleus leading to the transcriptional activation of antioxidant enzymes. In addition, the ALA-induced ROS production stimulates ERK phosphorylation and its downstream pathway to promote cell proliferation. Under HS conditions, HSPs genes including HSP70 are transcriptionally activated by HSF1. ALA promotes this process, leading to preservation of cellular homeostasis and reduction in cell damage. HS is also associated with disruption of the intestinal epithelial integrity, particularly by affecting E-cadherin, which is prevented by ALA. The ROS production is increased by HS exposure and leads not only to Nrf2 liberation and nuclear translocation resulting in upregulation of cytoprotective genes such as glutathione and HO-1. HS also stimulates the P38 MAPK and NF-kB pathways, thereby modulating the expression of TGF-β and COX-2. ALA inhibits the expression of HS-induced TGF-β and COX-2 mRNA expression. It remains to be further elucidated whether the preventive effects of ALA on the HS-induced expression and nuclear accumulation of Nrf2 is direct or indirect

With the current in vitro experiments, it could be demonstrated that in a Caco-2 cell model, the antioxidant ALA modulates not only the HS-induced oxidative stress response, but also prevents the disruption of intestinal barrier integrity by accelerating the reassembly of junctional complexes, hampering the delocalization of E-cadherin, and stimulating the intestinal epithelial healing, probably by enhancing the expression of HSP70 and preventing the fibrotic response induced by TGF-β.

These in vitro results provide additional insights in the mechanisms of action of ALA. Future in vivo experiments are needed to confirm the beneficial effects of dietary supplementation with ALA as a strategy to improve the resilience of animals and humans to hyperthermia-induced gastrointestinal injury.

## Electronic supplementary material

Below is the link to the electronic supplementary material.

Supplementary material 1 (PDF 187 KB)

Supplementary material 2 (PDF 192 KB)

Supplementary material 3 (PDF 440 KB)

Supplementary material 4 (PDF 380 KB)

## Abbreviations

AJ: Adherens junctions
ALA: α-Lipoic acid
Caco-2: Colorectal adenocarcinoma cells
COX-2: Cyclooxygenase-2
DHALA: Dihydrolipoic acid
ERK: Extracellular signal-related kinases
HS: Heat stress
HO-1: Heme oxygenase-1
HSF1: Heat shock factor-1
HSP: Heat shock protein
IkB: Inhibitor of kappa B
JC: Junctional complexes
Keap1: Kelch-like ECH-associated protein-1
LY: Lucifer yellow
MAPK: Mitogen-activated protein kinase
NF-kB: Nuclear factor kappa B
Nrf2: Nuclear factor erythroid 2-related factor 2
qRT-PCR: Quantitative real-time PCR
ROS: Reactive oxygen species
TEER: Transepithelial electrical resistance
TGF-β: Transforming growth factor-β
TJ: Tight junctions
