# Supplementary material for: α-Lipoic acid prevents the intestinal epithelial monolayer damage under heat stress conditions: model experiments in Caco-2 cells
Source: Eur J Nutr. 2017 Mar 27;57(4):1577–89. doi: 10.1007/s00394-017-1442-y (PMC5960005; doi:10.1007/s00394-017-1442-y)
Supplement: Supplementary file 1 — Supplementary material 1 (PDF 187 KB) [file 394_2017_1442_MOESM1_ESM.pdf]

Article title:

α-lipoic acid ameliorates the intestinal epithelial monolayer damage under heat stress conditions.

Journal name:

European Journal of Nutrition

Authors:

Soheil Varasteh, Johanna Fink-Gremmels, Johan Garssen, Saskia Braber

Corresponding author:

Dr. Saskia Braber

Utrecht University, Department of Pharmaceutical Sciences, Division of Pharmacology.

Universiteitsweg 99, 3584 CG, Utrecht, The Netherlands

Fax: +31(0)30 253 7900

Email: s.braber@uu.nl

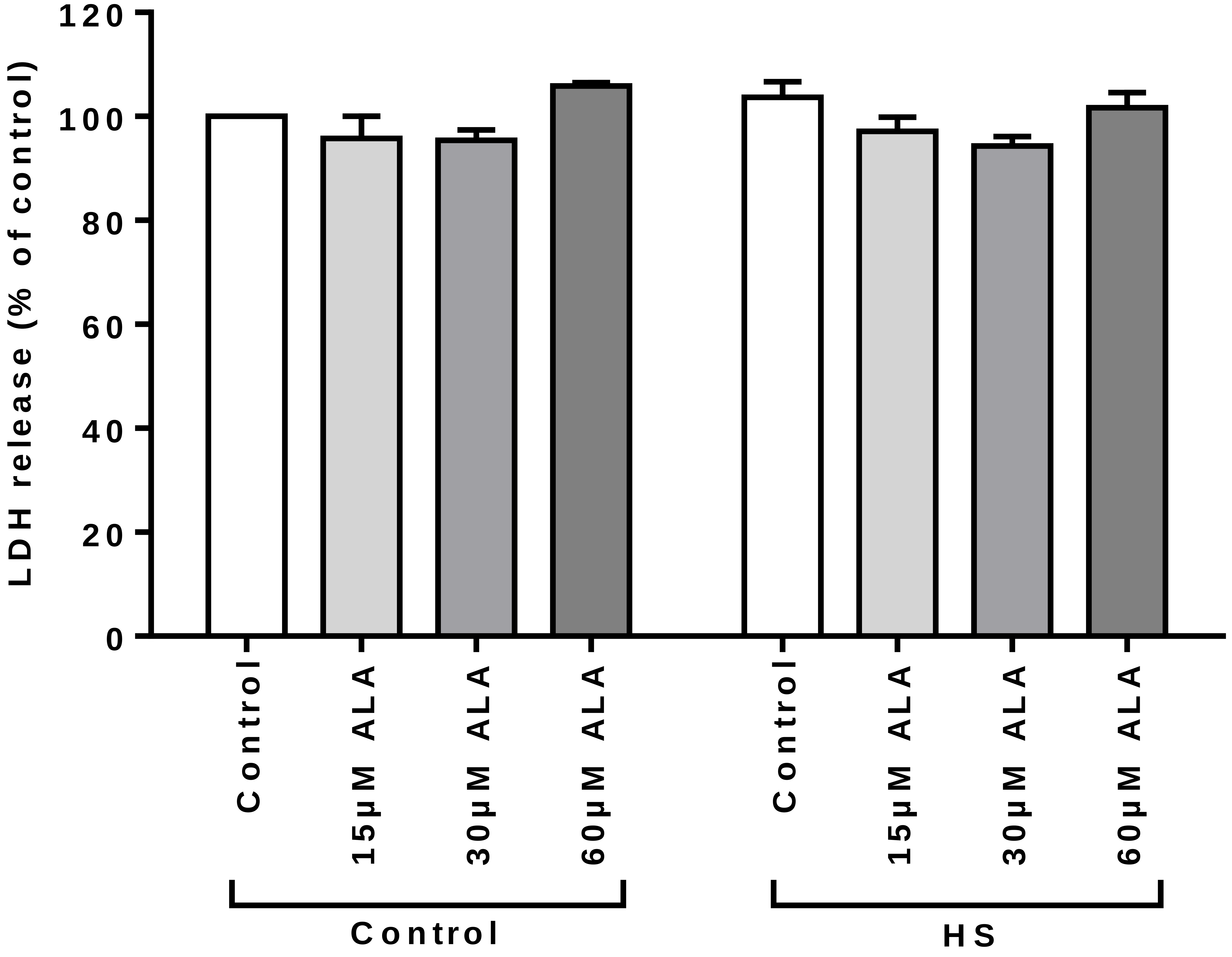

Supplementary Figure 1

Neither HS nor ALA treatment exerts a cytotoxic effect on Caco-2 cells. Caco-2 cells grown on inserts and pretreated with ALA (24h) were exposed to HS (42°C, 6h). Thereafter, the cytotoxic effect of HS (24h) and ALA treatment was determined by measuring the release of lactate dehydrogenase (LDH) in supernatants of the apical compartment of the inserts using the CytoTox 96 Non-Radioactive CytoToxicity Assay Kit (Promega Corporation, WI, USA) according to manufacturer's instructions. Results are expressed as LDH release relative to unstimulated cells as mean ± SEM of three independent experiments.
