# Supplementary material for: α-Lipoic acid prevents the intestinal epithelial monolayer damage under heat stress conditions: model experiments in Caco-2 cells
Source: Eur J Nutr. 2017 Mar 27;57(4):1577–89. doi: 10.1007/s00394-017-1442-y (PMC5960005; doi:10.1007/s00394-017-1442-y)
Supplement: Supplementary file 2 — Supplementary material 2 (PDF 192 KB) [file 394_2017_1442_MOESM2_ESM.pdf]

Article title:

α-lipoic acid ameliorates the intestinal epithelial monolayer damage under heat stress conditions.

Journal name:

European Journal of Nutrition

Authors:

Soheil Varasteh, Johanna Fink-Gremmels, Johan Garssen, Saskia Braber

Corresponding author:

Dr. Saskia Braber

Utrecht University, Department of Pharmaceutical Sciences, Division of Pharmacology.

Universiteitsweg 99, 3584 CG, Utrecht, The Netherlands

Fax: +31(0)30 253 7900

Email: s.braber@uu.nl

Supplementary Figure 2. Primer sequences used for qRT-PCR

Gene	Primer sequence (5'-3')		AT	References
	Forward	Reverse		
**β-Actin**	CTGGAACGGTGAAGGTGACA	AAGGGACTTCCTGTAACAATGCA	63	NM_001101
**HSP70**	AGAGCCGAGCCGACAGAG	CACCTTGCCGTGTTGGAA	57	NG_011855.1
**Nrf2**	CAGGTTGCCCACATTCCCAAATCA	AGCAATGAAGACTGGGCTCTCGAT	60	NM_001145413.2
**TGF-β**	CACGTGGAGCTGTACCAGAA	GAACCCGTTGATGTCCACTT	60	NM_000660.5
**COX-2**	GGAACACAACAGAGTATGCG	AAGGGGATGCCAGTGATAGA	60	NM_000963.3
**HO-1**	GCCACCAAGTTCAAGCAGCT	CAGTGCCCACGGTAAGGAAG	61.2	NM_002133.2
