# Supplementary material for: α-Lipoic acid prevents the intestinal epithelial monolayer damage under heat stress conditions: model experiments in Caco-2 cells
Source: Eur J Nutr. 2017 Mar 27;57(4):1577–89. doi: 10.1007/s00394-017-1442-y (PMC5960005; doi:10.1007/s00394-017-1442-y)
Supplement: Supplementary file 3 — Supplementary material 3 (PDF 440 KB) [file 394_2017_1442_MOESM3_ESM.pdf]

Article title:

α-lipoic acid ameliorates the intestinal epithelial monolayer damage under heat stress conditions.

Journal name:

European Journal of Nutrition

Authors:

Soheil Varasteh, Johanna Fink-Gremmels, Johan Garssen, Saskia Braber

Corresponding author:

Dr. Saskia Braber

Utrecht University, Department of Pharmaceutical Sciences, Division of Pharmacology.

Universiteitsweg 99, 3584 CG, Utrecht, The Netherlands

Fax: +31(0)30 253 7900

Email: s.braber@uu.nl

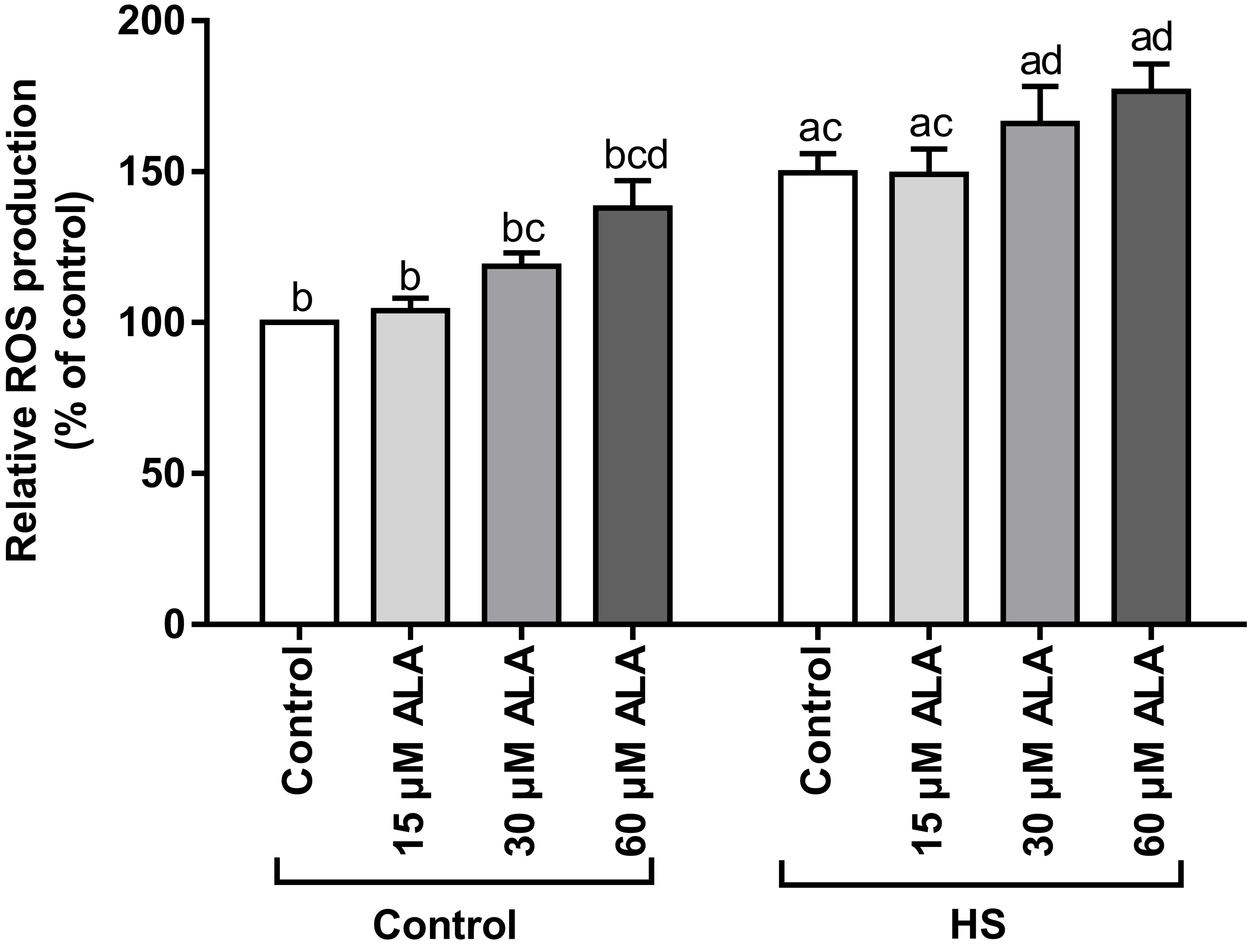

Supplementary Figure 3

HS increases ROS production. Caco-2 cells grown on 96-well plate (17 days) were pretreated with different ALA concentrations for 24h. After washing with pre-warmed PBS, cells were incubated with DMEM supplemented with 10 µM DMSO-dissolved H2DCFDA (Life Technologies, NY, USA) for 1h. Thereafter, cells were washed, incubated with ALA and exposed to 37°C or 42°C for 6h. Relative ROS production was quantified immediately before HS exposure and 6h after HS by a fluorometer (FLUOstar OPTIMA, Offenburg, Germany) at excitation and emission wavelengths of 490 nm and 525 nm). Results are expressed as ROS production normalized with initial ROS values relative to unstimulated cells. Data are expressed as mean ± SEM of three independent experiments. Different lower-case denote significant differences among groups.
