# Supplementary material for: α-Lipoic acid prevents the intestinal epithelial monolayer damage under heat stress conditions: model experiments in Caco-2 cells
Source: Eur J Nutr. 2017 Mar 27;57(4):1577–89. doi: 10.1007/s00394-017-1442-y (PMC5960005; doi:10.1007/s00394-017-1442-y)
Supplement: Supplementary file 4 — Supplementary material 4 (PDF 380 KB) [file 394_2017_1442_MOESM4_ESM.pdf]

Article title:

α-lipoic acid ameliorates the intestinal epithelial monolayer damage under heat stress conditions.

Journal name:

European Journal of Nutrition

Authors:

Soheil Varasteh, Johanna Fink-Gremmels, Johan Garssen, Saskia Braber

Corresponding author:

Dr. Saskia Braber

Utrecht University, Department of Pharmaceutical Sciences, Division of Pharmacology.

Universiteitsweg 99, 3584 CG, Utrecht, The Netherlands

Fax: +31(0)30 253 7900

Email: s.braber@uu.nl

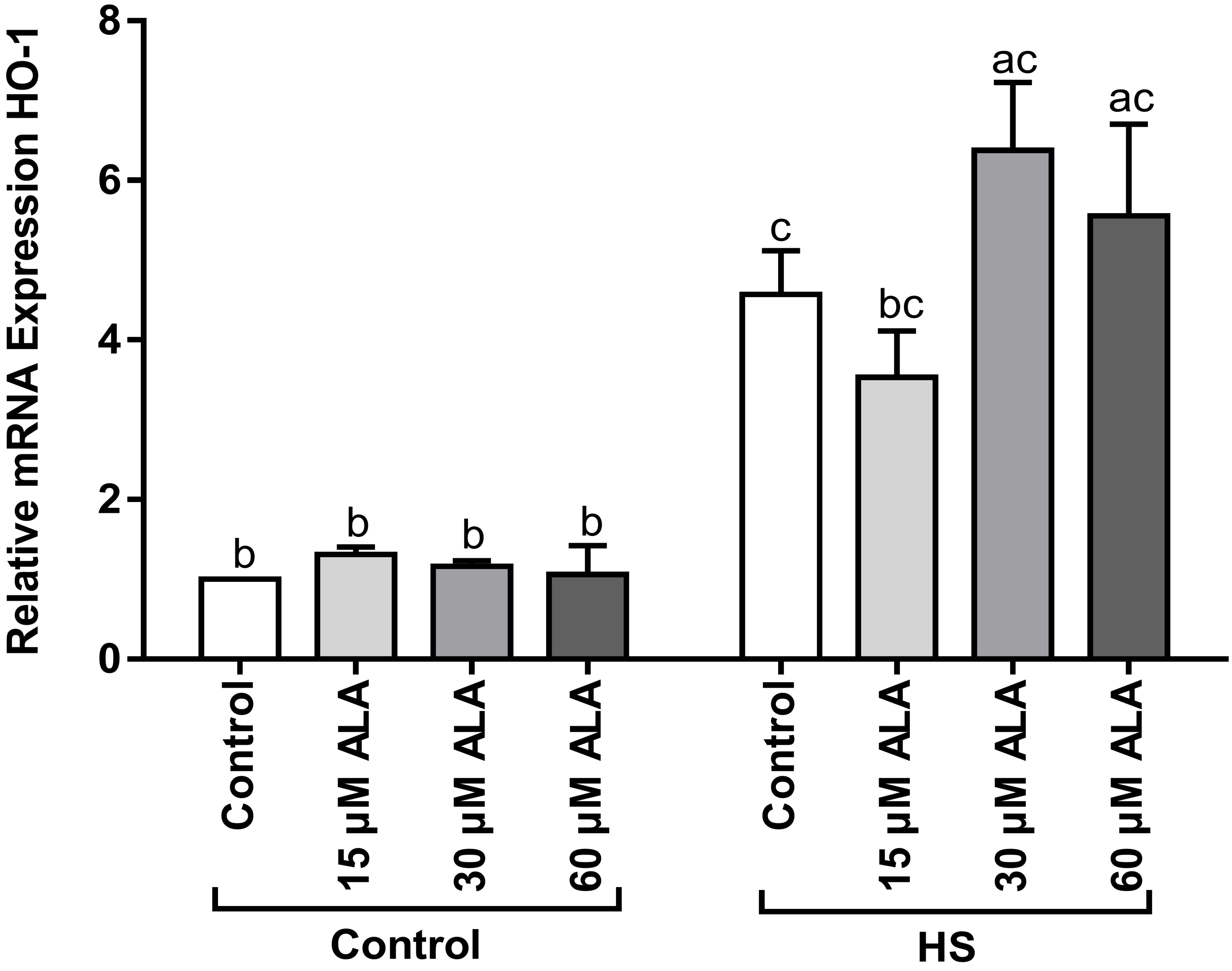

Supplementary Figure 4

HO-1 mRNA expression is increased after HS exposure. Caco-2 cells grown on inserts and pretreated with ALA (24h) were exposed to HS (42°C) for 6h (qRT-PCR) to evaluate the expression of HO-1 in mRNA level. Results are expressed as mRNA expression (normalized with β-actin) relative to unstimulated cells as mean ± SEM of three independent experiments. Different lower-case denote significant differences among groups.
